# Cryptic fungal diversity revealed in deep-sea sediments associated with whale-fall chemosynthetic ecosystems

**DOI:** 10.1080/21501203.2020.1799879

**Published:** 2020-08-06

**Authors:** Yuriko Nagano, Toshiko Miura, Taishi Tsubouchi, Andre O. Lima, Masaru Kawato, Yoshihiro Fujiwara, Katsunori Fujikura

**Affiliations:** a Deep-Sea Biodiversity Research Group, Marine Biodiversity and Environmental Assessment Research Center, Research Institute for Global Change (RIGC), Japan Agency for Marine-Earth Science and Technology (JAMSTEC), Yokosuka, Japan; b Research Institute of Environment, Agriculture and Fisheries, Osaka, Japan; c Department of Bacteriology, Graduate School of Medicine, Public University Corporation Osaka City University (OCU), Osaka, Japan; d Department of Biological Sciences, University of Vale do Itajaí (UNIVALI), Itajaí, Brazil

**Keywords:** Early diverging fungi, Ion Torrent, Kagoshima Bay, marine environment, São Paulo Ridge, whalebone

## Abstract

In this study, sediments from whale-fall chemosynthetic ecosystems (two different sites, one naturally occurring at 4200 m water depth in South Atlantic Ocean and one artificially immersed at 100 m water depth in Kagoshima Bay, Japan) were investigated by Ion Torrent PGM sequencing of the ITS region of ribosomal RNA to reveal fungal communities in these unique marine environments. As a result, a total of 107 (897 including singletons) Operational Taxonomic Units (OTUs) were obtained from the samples explored. Composition of the 107 OTUs at the phylum level among the five samples from two different whale-fall sites was assigned to Ascomycota (46%), Basidiomycota (7%), unidentified fungi (21%), non-fungi (10%), and sequences with no affiliation to any organisms in the public database (No-match) (16%). The high detection of the unidentified fungi and unassigned fungi was revealed in the whale-fall environments in this study. Some of these unidentified fungi are allied to early diverging fungi and they were more abundant in the sediments not directly in contact with whalebone. This study suggests that a cryptic fungal community exists in unique whale-fall ecosystems.

## Introduction

Fungi are major ecological players in both terrestrial and aquatic environments that cycle organic matter and channel nutrients across trophic levels. High-throughput sequencing studies of fungal communities are redrawing the map of the fungal kingdom by hinting at its enormous and largely uncharted taxonomic and functional diversity (Nilsson et al. 2019). The presence of fungi in deep-sea environments with their taxonomic novelty and ecological importance in ecosystems is recently recognised with much interest. Many fungi have been isolated and reported from various deep-sea environments, including deep marine subsurface from more than 50 years ago (Roth et al. 1964; Kohlmeyer 1969, 1977; Raghlukumar et al. 2004; Burgaud et al. 2009, 2010, 2016; Damare et al. 2006; Nagahama et al. 2008; Dupont et al. 2009; Le Calvez et al. 2009; Nagano and Nagahama 2012; Singh et al. 2012; Redou et al. 2015; Nagano et al. 2016, 2019; Wei et al. 2018). Culture-independent environmental DNA-based techniques, especially using high-throughput sequencing, revealed comprehensive fungal diversity, including many novel fungal phylotypes (Bass et al. 2007; Lai et al. 2007; Nagano et al. 2010, 2017; Edgcomb et al. 2011; Singh et al. 2011, 2012; Orsi et al. 2013; Redou et al. 2014; Xu et al. 2014, 2018, 2019; Zhang et al. 2014, 2016; Barone et al. 2018; Vargas-Gastelum et al. 2019). Novel phylotypes affiliated with basal fungal lineages have been abundantly detected, especially in unique deep-sea chemosynthetic ecosystems, such as hydrothermal vents and hydrocarbon seeps (Le Calvez et al. 2009; Nagahama et al. 2011; Xu et al. 2017).

Sunken whale carcases, so-called “whale-falls”, are another type of sulfogenic habitat that supports chemosynthetic communities in deep-sea environments similar to hydrocarbon seeps and hydrothermal vent systems. Whale-falls produces unique organic and sulphide-rich habitat islands at the seafloor. Giant body sizes and especially high bone-lipid content allow great whale carcases to support a sequence of heterotrophic and chemosynthetic microbial assemblages in the energy-poor deep sea (Smith et al. 2015; Onishi et al. 2020). Since the first recognition of the whale-fall chemosynthetic ecosystems in deep sea off California by Smith et al. (1989), it is known that metazoan communities in whale-fall ecosystems contain many new species and evolutionary novelties, including bone-eating worms and snails with faunal overlap with other deep-sea chemosynthetic communities, such as hydrothermal vents, cold seeps and wood falls (Fujiwara et al. 2010; Sumida et al. 2016). Prokaryotic communities in whale-fall chemosynthetic environments have been well investigated and documented (Deming et al. 1997; Smith et al. 1998; Goffredi et al. 2008; Miyazaki et al. 2008, 2010; Goffredi and Orphan 2010; Danise et al. 2012; Cavalett et al. 2017). However, to the best of our knowledge, one of the most important component groups of organisms in many ecosystems, fungi, has never been investigated in whale-fall chemosynthetic environments.

The aim of this study was to investigate fungal diversity in sediments associated with whale-fall chemosynthetic ecosystems in order to increase our knowledge of fungal communities in deep-sea environments, especially in unique chemosynthetic ecosystems. In this study, sediments of whale-fall chemosynthetic ecosystems from two different sites, one naturally occurring at 4200 m water depth in South Atlantic Ocean (Sumida et al. 2016) and one artificially immersed at 100 m water depth in Kagoshima Bay, Japan (Fujiwara et al. 2007; Tsubouchi et al. 2015) were investigated by Ion Torrent PGM targeting ITS region of ribosomal RNA to reveal fungal community in these unique marine environments.

## Materials and methods

### Site description and sediment sampling

**Figure 1.**
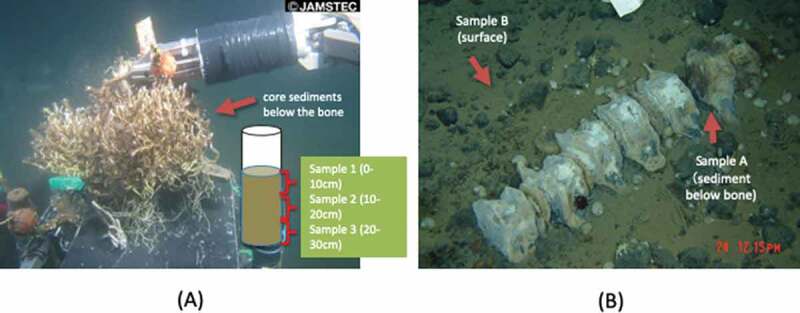
Whale-fall sites investigated in this study. (a) Kagoshima Bay, Japan (Water depths = 101 m) (b) The Sao Paulo Ridge, off Brazil (Water depths = 4,204 m).

Five sediment samples were collected at two geologically and physiologically different whale-fall chemosynthetic sites. Three sediment samples were collected as a core sample at Kagoshima Bay, Japan (Site 1: 31°39.746ʹ N, 130°48.050ʹ E, Water depths = 101 m) during the NT12-09 cruise with the remotely operated vehicle (ROV) *Hype-Dolphin* (Dive no. 1368) operated on 12 April 2012. The sediment core sample was collected from just below the whalebone, which was heavily colonised by tubeworms (*Lamellibrachia satsuma*) (Figure 1(a)), and cut into layers at different depths (Sample ID, 1: 0–5 cm, 2: 5–10 cm, 3: 10–15 cm) from the surface of the seafloor. The whalebone at this site was artificially immersed for research purposes during the NT05-12 leg1 cruise on 28 July 2005 and placed for almost 7 years until sampled in 2012. The other two sediment samples were collected at the São Paulo Ridge, off Brazil (Site 2: 28° 31.1191ʹ S, 41° 39.4097ʹ W, Water depths = 4,204 m) during the YK13-04 leg1 of the *Iata-piuna* cruise by using the human-occupied vehicle (HOV) *Shinkai 6500* operated on 23 April 2013. Surface sediment samples were collected from just below the whalebone (Sample ID: A) and approximately one metre away from the bone (Sample ID: B) (Figure 1(b)). Whalebones at this site were found as the first record of a natural whale fall in the deep Atlantic Ocean and as the deepest record to date (Sumida et al. 2016). Details of the collected samples are described in Table 1.

**Table 1.** Details of the whale-fall sediment samples examined in this study.

Sample name	Date of sampling	Locality area	Latitude	Longitude	Water Depth (m)	Depth below the seafloor (cm)	Whale-fall type	Remarks
1	2012.4.12	Kagoshima Bay, Japan	31°39.746ʹ N	130°48.050ʹ E	101	0–5	Artificially immersed	Core sediment below the whale bone
2	2012.4.12	Kagoshima Bay, Japan	31°39.746ʹ N	130°48.050ʹ E	101	5–10	Artificially immersed	Core sediment below the whale bone
3	2012.4.12	Kagoshima Bay, Japan	31°39.746ʹ N	130°48.050ʹ E	101	10–15	Artificially immersed	Core sediment below the whale bone
A	2013.4.23	Sao Paulo Ridge, Off Brazil	28°31.1191ʹ S	41°39.4097ʹ W	4204	Surface	Naturally occurred	Surface sediment below the whale bone
B	2013.4.23	Sao Paulo Ridge, Off Brazil	28°31.1191ʹ S	41°39.4097ʹ W	4204	Surface	Naturally occurred	Surface sediment outside whale bone

### DNA extractions, PCR amplifications and sequencing

DNA was extracted from 0.5 g of each sediment sample by the employment of ISOIL for beads beating kit (Nippon Gene, Japan), in accordance with the manufacturer’s instructions. Extracted DNA was stored at −20°C, prior to PCR amplification. For extractions, a negative extraction control containing all reagents minus sediment was performed. Fungal DNA was amplified with the primer set ITS-1FS (5ʹ-CTTGGTCATTTAGAGGAAGTAA-3ʹ)/ITS4 (5ʹ-TCCTCCGCTTATTGATATGC-3ʹ) as a primary primer set and ITS1 (5ʹ-TCCGTAGGTGAACCTGCGG-3ʹ)/ITS2 (5ʹ-GCTCCGTTCTTCATCGATGC-3ʹ) as a nested primer set (White et al. 1990; Gardes and Bruns 1993). PCR reaction mixes (20 µl) contained: 10 µl of SYBR Premix Ex Taq (TaKaRa, Japan), 0.4 µM (each) of a pair of primers and 1–2 µl of DNA template (10–100 ng). For the nested PCR, 0.5 µl of primary PCR product was used as a DNA template. The 7500 Real-Time PCR System (Applied Biosystems) was used to determine the optimal cycle number by reference to cycle threshold (Ct) values for Ion Torrent PGM analysis. The real-time PCR conditions used were 95°C for 30 sec, 40 cycles of 95°C for 5 sec, 60°C for 34 sec, and 95°C for 15 sec, followed by 60°C for 60 sec. Ct values were defined as the number of cycles required for normalised fluorescence to reach a manually set threshold of 20% total fluorescence. PCR amplification was performed in a GeneAmp® PCR system 9700 (Applied Biosystems) with calculated Ct value, which was 19 for all samples, with the same conditions as the real-time PCR. The PCR products were purified using the Agencourt AMPure XP Reagent (Beckman Coulter, Brea, CA, USA). The purified PCR amplicons were end-repaired using the Ion Plus Fragment Library Kit (Life Technologies Inc., Grand Island, NY, USA), following the manufacturer’s protocol. The end-repaired amplicons were purified using the Agencourt AMPure XP Reagent. Sequencing adapters with the sample identification barcoding key were ligated using an Ion Xpress Fragment Library Kit, following the manufacturer’s protocol. The adapter-ligated and nick-translated amplicons were purified using the Agencourt AMPure XP Reagent. The concentrations of the prepared libraries were determined by quantitative PCR using the Ion Library Quantitation Kit (Life Technologies Inc.). The amount of library required for template preparation was calculated using the template dilution factor calculation described in the protocol. Diluted libraries were pooled for library amplification using the Ion One Touch and ES systems (Life Technologies Inc.). Emulsion PCR to incorporate the library to the sequencing beads was performed using the Ion OneTouch instrument with an Ion OneTouch OT2 400 Kit (Life Technologies Inc.). Finally, the library sample was sequenced on an Ion Torrent Personal Genome Machine using an Ion 318 chip and the Ion PGM 400 sequencing Kit (Life Technologies Inc.), following the manufacturer’s protocols. The raw sequence data (.fastq file) are available in the DNA Data Bank of Japan (DDBJ) under accession number DRA010220.

### Data processing and analyses

The sequence data were analysed using the Mothur pipeline (v. 1.32.1) following a modified standard operating procedure (Schloss et al. 2009, 2011). In brief, the data were subjected to quality control, whereby each sequence was screened for a match to the sequencing primer and thresholds for average-Phred quality score (Q ≥ 20), ambiguous bases (count = 0), and homopolymers (length ≤8). Sequences shorter than 100 bp after quality trimming were not considered. All potentially chimeric sequences were identified using Mothur-embedded UCHIME (chimera.uchime) (Edgar et al. 2011) and were removed. The sequence dataset was normalised to 40,703 sequences per sample (the smallest sample size) to reduce bias associated with different numbers of reads in the different samples (Gihring et al. 2012). Unique sequences were pairwise aligned (Needlema and Wunsch 1970) and the resultant distance matrix clustered into operational taxonomic units (OTUs) using the nearest neighbour algorithm at >97% similarity. Singleton OTUs (*n* = 790) were removed as most next-generation sequencing (NGS) singletons are considered to be artefacts (Tedersoo et al. 2010). Classification of the sequences was performed using the UNITE + International Nucleotide Sequence Databases (INSD: NCBI, EMBL, DDBJ) ITS reference database (v.6; released on 10 September 2014) with the BLASTn algorithm (Abarenkov et al. 2010). Results were then confirmed by using the top-100 best BLASTn analyses, which were performed manually in 2019 (https://blast.ncbi.nlm.nih.gov/). Some results were modified when confirmed by manual analysis. Sequences, which the majority (>80%) of top-100 BLASTn analyses showed similarity (even though with low query coverage) to non-fungal organisms, were treated as “non-fungi”. In the same way, sequences affiliated with uncultured fungi or unidentified fungi without lower taxonomic level classification were treated as “unidentified fungi”. Sequences with no affiliation to any organisms were treated as “non-match”. The diversity of fungal communities in each sample was compared using multiple metrics for rarefaction, observed OTU richness, Good’s coverage (complement of the ratio between local singleton OTUs and the total sequence count)(Good and Toulmin 1956), Simpson diversity index (1/D) (Simpson 1949), Shannon diversity index (H) (Shannon 1948), calculated in Mothur software package. Community similarities across the samples were visualised using nonmetric multidimensional scaling (NMDS) based on the Morisita-horn metrics in R (R Core Team 2015). NMDS was selected as a preferred ordination procedure because it makes few assumptions about the distribution of the species. Morisita-horn metrics was chosen because sample size and diversity of the sample have little influence in its calculation (Morisita 1959; Wolda 1981) and these methods are commonly used in microbial ecology studies (Tedersoo et al. 2014). Ordination plots were created using the “metaMDS” function in the R vegan package (Oksanen et al. 2013), which incorporated relative abundance (transformation first square-root then Wisconsin double standardisation) or presence–absence of OTU data.

## Results

### Sequence analysis

A total of 3,123,023 sequences with an average read length of 157 bp were generated by an Ion Torrent PGM for 5 sediment samples collected from two different whale-fall chemosynthetic sites. After quality control, a total of 401,404 sequences (13%) were used for analysis. The numbers of sequences for each sample are listed in Table 2. Clustering at 97% identity produced 897 unique OTUs across the 5 sediment samples, of which 790 OTUs were singletons. The remaining 107 OTUs were used in further analyses. Good’s coverage was higher than 99.9% (Table 2) throughout the samples. This data indicates an excellent overall OTU coverage afforded by the deep sequencing.

**Table 2.** Details of the obtained sequence reads, fungal OTU richness, coverage, and diversity indices in each sample.

Sample Name	Sequence data filtering				Diversity			
	No. of reads			% of high quality reads	Number of observed OTUs (97%)	Good’s coverage (%)	Shannon (H)	Simpson (1/*D*)
	Before filtering		After filtering					
1	927,048		158,426	17.09	20	99.98%	0.08	1.02
2	490,476		58,963	12.02	67	100.00%	2.68	10.04
3	527,403		85,766	16.26	25	99.98%	0.47	1.28
A	520,567		57,546	11.05	43	99.99%	1.74	3.86
B	657,529		40,703	6.19	38	99.98%	0.78	2.03

### Fungal diversity and communities

**Figure 2.**
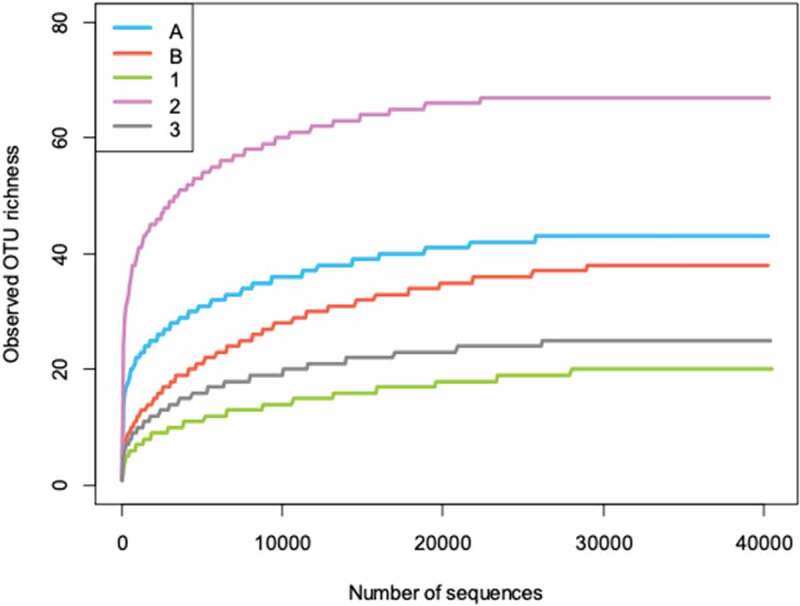
Rarefaction curves of the observed fungal OTU richness at 97% sequence similarity in each sample.

**Figure 3.**
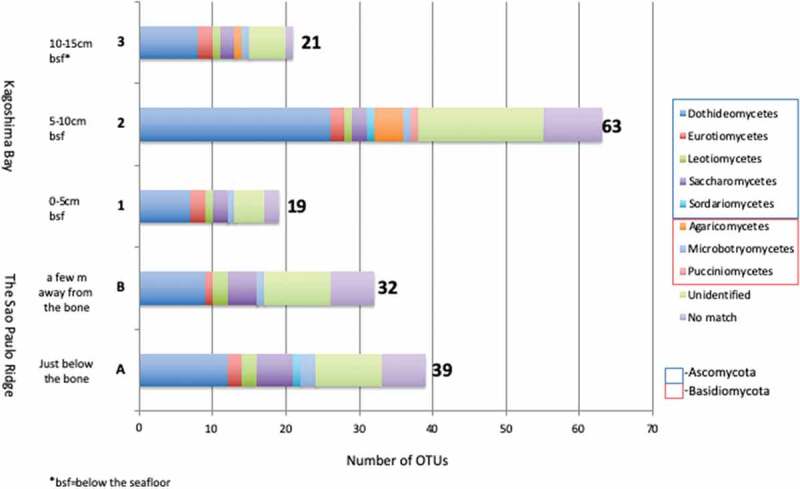
Proportion of detected OTUs assigned to class level in each sample.

Rarefaction curves were shown in Figure 2 and a number of observed OTUs in each sample are shown in Table 2 (including non-fungal OTUs) and Figure 3 (excluding non-fungal OTUs). The highest number of OTUs (67 OTUs: 63 fungal and 4 non-fungal) was obtained in sample 2, followed by sample A (43 OTUs: 39 fungal and 4 non-fungal), sample B (38 OTUs: 32 fungal and 6 non-fungal), and sample 3 (25 OTUs: 21 fungal and 4 non-fungal). The lowest number of OTUs (20 OTUs: 19 fungal and 1 non-fungal) was observed in sample 1. Both Shannon and Simpson diversity indices showed the highest diversity in sample 2 and the lowest diversity in sample 1.

**Figure 4.**
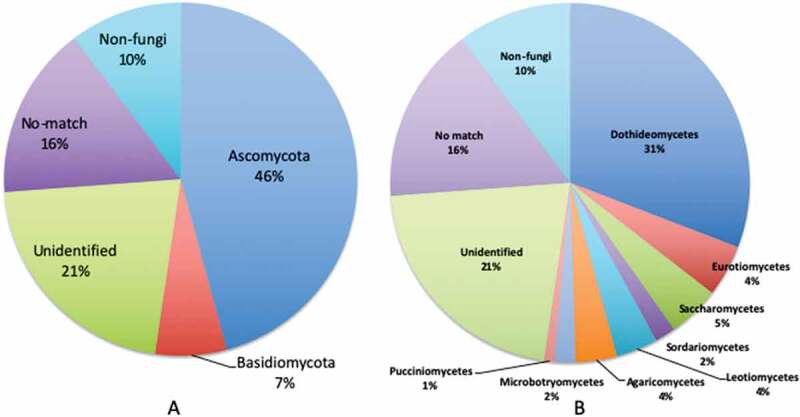
Proportion of obtained 107 OTUs assigned to (a) phylum level (b) class level.

A total of 107 OTUs were recovered from 5 marine sediments associated with whale-fall chemosynthetic ecosystems. Composition of the 107 OTUs at the phylum level among the 5 samples from two different sites of whale fall was assigned to Ascomycota (46%), Basidiomycota (7%), unidentified fungi (21%), non-fungi (10%) and sequences with no affiliation to any organisms in the public database (No-match)(16%)(Figure 4(a)). Classification of the observed 96 fungal OTUs (11 non-fungal OTUs were excluded, OTU16, 26, 33, 47, 67, 68, 71, 87, 90, 93, 94) are shown in Table 3. The majority of non-fungal OTUs showed similarity to Metazoa, such as deep-sea marine arrow worms, siphonophores and cnidarians, but most are with very low query coverage. Assignable fungal OTUs were dominated by Dothideomycetes (31%), followed by Saccharomycetes (5%), Eurotiomycetes (4%), Leotiomycetes (4%), Agaricomycetes (4%), Sordariomycetes (2%), Microbotryomycetes (2%) and Pucciniomycetes (1%) (Figure 4(b)). The most frequently detected OTU genera were *Paraconiothyrium* (4 OTUs) and *Phoma* (4 OTUs), followed by *Candida* (2), *Exophiala* (2), *Lycoperdon* (2), *Paraphaeosphaeria* (2), *Penicillium* (2) and *Rhodosporidium* (2). The top-ten most abundant OTUs were *Leptosphaeria* sp. (23.5% of the sequences), unidentified fungi (17.9%), unidentified fungi (11.0%), DSF-Group1 (10.5%), *Rhodosporidium diobovatum* (8.6%), *Cyclothyrium* sp. (4.2%), *Periconia* sp. (3.4%), unidentified fungi (3.3%), *Phoma* sp. (2.7%), and unidentified fungi (2.5%). The majority of OTUs (*n* = 82 out of 96) accounts for less than 1.0% of the sequences (Table 3). Classification of OTUs revealed that 35 out of 96 (36.5%) fungal OTUs exhibited >98% sequence similarity, and 46 (48.0%) exhibited >97% similarity, to pre-existing ITS sequences in public databases.

**Table 3.** Classification of obtained 107 OTUs with overall abundance. Phylum rank: (A: Ascomycetes, B: Basidiomycetes, U: unidentified, -: No match).

OTU No.	Phylum	Class	Species	Accession No.	Identity(%)	Coverage(%)	Overall abundance(%)
1	A	Dothideomycetes	*Leptosphaeria* sp.	AB693792	98	100	23.502
2	U	Unidentified	Uncultured fungus	HM030613	97	30	17.882
3	U	Unidentified	Uncultured fungus	HM240101	98	18	11.010
4	A	Saccharomycetes	Uncultured fungus (DSF-G1)	KJ194363	97	100	10.451
5	B	Microbotryomycetes	*Rhodosporidium diobovatum*	JQ993385	99	100	8.566
6	A	Dothideomycetes	*Cyclothyrium* sp.	KP309921	88	74	4.152
7	A	Dothideomycetes	*Periconia* sp.	JX868735	97	100	3.354
8	U	Unidentified	Uncultured fungus	JX915310	100	29	3.284
9	A	Dothideomycetes	*Phoma* sp.	KT199712	100	100	2.747
10	U	Unidentified	Uncultured fungus	JN905953	100	20	2.530
11	U	Unidentified	Uncultured fungus	AB615563	94	88	1.469
12	A	Leotiomycetes	*Ciboria shiraiana*	JN033430	99	100	1.087
13	A	Dothideomycetes	*Phoma multirostrata*	KJ686366	100	100	1.041
14	A	Dothideomycetes	*Pleosporales* sp.	HQ631052	99	100	0.960
15	A	Dothideomycetes	*Paraphaeosphaeria angularis*	JX496047	95	100	0.912
17	A	Saccharomycetes	*Candida* sp.	AJ549823	98	100	0.740
18	A	Dothideomycetes	*Pleosporales* sp.	HQ631002	96	100	0.726
19	U	Unidentified	Uncultured fungus	JF945481	97	19	0.631
20	A	Eurotiomycetes	*Penicillium citreonigrum*	LN808957	99	100	0.464
21	U	Unidentified	Uncultured fungus	AB507841	95	24	0.433
22	A	Dothideomycetes	*Microsphaeropsis arundinis*	EU664487	98	100	0.344
23	-	-	-	-	-	-	0.257
24	A	Dothideomycetes	*Capnodium* sp.	HQ631045	98	100	0.241
25	U	Unidentified	Uncultured fungus	FJ626929	83	42	0.228
27	-	-	-	-	-	-	0.196
28	A	Leotiomycetes	*Leohumicola* sp.	AF461659	90	100	0.155
29	U	Unidentified	Uncultured fungus	JX974807	97	27	0.151
30	B	Agaricomycetes	*Melanotus caricicola*	AY129365	93	100	0.149
31	A	Dothideomycetes	*Pleosporales* sp.	AB809634	76	100	0.130
32	-	-	-	-	-	-	0.125
34	A	Saccharomycetes	Uncultured fungus G57 (DSF-G1)	DQ279844	83	100	0.096
35	A	Dothideomycetes	*Pleosporales* sp.	HQ631052	88	100	0.092
36	-	-	-	-	-	-	0.091
37	A	Eurotiomycetes	*Exophiala equina*	JX681045	99	100	0.078
38	A	Dothideomycetes	*Teichospora melanommoides*	KU601585	92	100	0.065
39	B	Agaricomycetes	*Gyrodontium sacchari*	KR867661	98	100	0.062
40	-	-	-	-	-	-	0.042
41	A	Dothideomycetes	*Phaeosphaeria oryzae*	KM434269	97	100	0.039
42	A	Dothideomycetes	*Pleosporales* sp.	HQ696074	99	99	0.034
43	B	Agaricomycetes	*Lycoperdon perlatum*	KP340200	99	100	0.030
44	A	Dothideomycetes	*Paraconiothyrium brasiliense*	JQ936270	99	100	0.028
45	U	Unidentified	Uncultured fungus	JN904206	93	22	0.025
46	A	Dothideomycetes	*Hortaea werneckii*	KP341543	99	100	0.022
48	A	Dothideomycetes	*Pleosporales* sp.	KP269045	99	100	0.021
49	A	Eurotiomycetes	*Aspergillus pseudoglaucus*	KP670428	98	100	0.019
50	A	Leotiomycetes	*Cadophora malorum*	KF053555	96	100	0.014
51	-	-	-	-	-	-	0.011
52	A	Dothideomycetes	*Paraphaeosphaeria michotii*	JX629110	91	100	0.011
53	A	Dothideomycetes	*Paraconiothyrium hawaiiense*	KF498872	99	100	0.010
54	U	Unidentified	Uncultured fungus	KC966068	97	100	0.010
55	U	Unidentified	Uncultured fungus	KC491368	89	100	0.008
56	U	Unidentified	Uncultured fungus	JX387630	100	9	0.008
57	U	Unidentified	Uncultured fungus	JQ666760	84	35	0.007
58	U	Unidentified	Uncultured fungus	AB507841	85	96	0.007
59	U	Unidentified	Uncultured fungus	JF94548	97	20	0.006
60	U	Unidentified	Uncultured fungus	JX345842	100	21	0.006
61	-	-	-	-	-	-	0.005
62	-	-	-	-	-	-	0.005
63	-	-	-	-	-	-	0.004
64	A	Dothideomycetes	*Lophiostoma corticola*	EU770246	92	98	0.004
65	A	Dothideomycetes	*Paraconiothyrium hawaiiense*	EU715661	98	100	0.004
66	U	Unidentified	Uncultured fungus	FJ265946	100	19	0.004
69	A	Dothideomycetes	*Pleosporales* sp.	HE584879	88	97	0.003
70	U	Unidentified	Uncultured fungus	KC491368	94	100	0.003
72	-	-	-	-	-	-	0.002
73	-	-	-	-	-	-	0.002
74	-	-	-	-	-	-	0.002
75	A	Dothideomycetes	*Coniothyrium glycines*	KF251211	88	100	0.002
76	-	-	-	-	-	-	0.001
77	-	-	-	-	-	-	0.001
78	-	-	-	-	-	-	0.001
79	U	Unidentified	Uncultured fungus	KC215961	100	10	0.001
80	A	Eurotiomycetes	*Exophiala xenobiotica*	JX681049	98	100	0.001
81	B	Agaricomycetes	*Lycoperdon perlatum*	KF551247	98	100	0.001
82	A	Dothideomycetes	*Arthopyreniaceae* sp.	KC218451	95	100	0.001
83	B	Pucciniomycetes	*Thekopsora areolata*	KJ546897	97	14	0.001
84	A	Saccharomycetes	Uncultured fungus G57 (DSF-G1)	DQ279844	78	96	0.001
85	-	-	-	-	-	-	0.001
86	A	Sordariomycetes	*Periconia* sp.	JX868735	91	100	0.001
88	-	-	-	-	-	-	0.001
89	A	Sordariomycetes	*Gibellulopsis* sp.	KC287233	94	97	0.001
91	A	Dothideomycetes	*Perisporiopsis* sp.	HM031459	95	100	0.001
92	A	Saccharomycetes	*Candida* sp.	AJ549823	95	100	0.001
95	A	Leotiomycetes	*Helotiales* sp.	JX507731	93	34	0.001
96	U	Unidentified	Uncultured fungus	JX387630	94	13	0.001
97	B	Microbotryomycetes	*Rhodosporidium diobovatum*	KP329705	95	100	0.001
98	A	Eurotiomycetes	*Penicillium corylophilum*	AF033450	95	100	0.001
99	A	Dothideomycetes	*Camarosporium psoraleae*	KF777143	98	71	0.001
100	A	Dothideomycetes	*Periconia* sp.	JX868735	92	100	0.001
101	A	Dothideomycetes	*Peyronellaea combreti*	KJ869134	95	100	0.001
102	A	Dothideomycetes	*Phoma herbarum*	KF251212	95	100	0.001
103	A	Dothideomycetes	*Periconia* sp.	KP890580	93	100	0.001
104	A	Dothideomycetes	*Phoma herbarum*	KF251212	93	100	0.001
105	U	Unidentified	Uncultured fungus	JF945688	84	72	0.001
106	U	Unidentified	Uncultured fungus	JF945688	86	61	0.001
107	A	Dothideomycetes	*Paraconiothyrium brasiliense*	JQ936270	96	100	0.001

**Figure 5.**
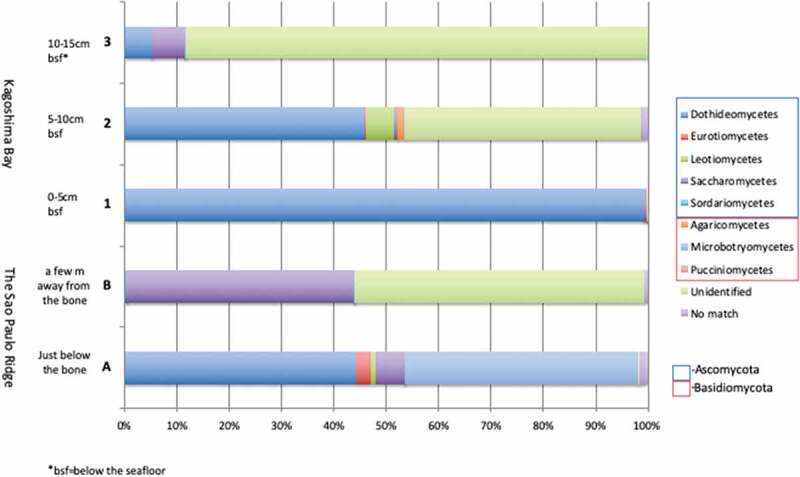
Abundance of detected fungal sequences at the class level in each sample.

Distribution and abundance of fungal OTUs at the class level in each sample are shown in Figure 5.

**Figure 6.**
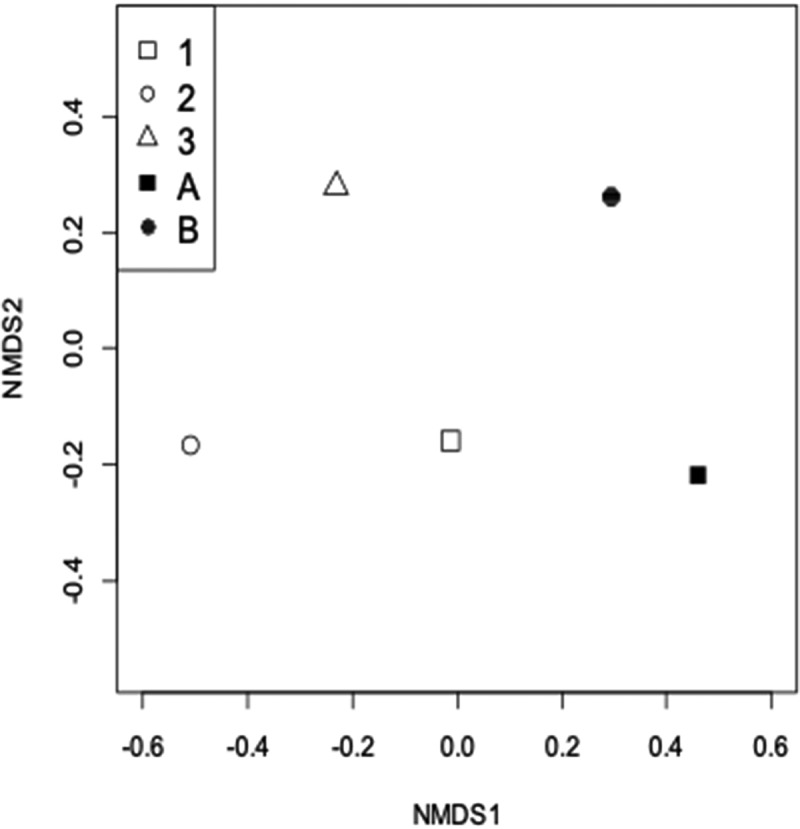
NMDS ordination of the fungal community structure.

Although the composition of fungal communities throughout all five samples showed a similar pattern (Figure 3), the abundance of each fungal class showed a significant difference in each sample (Figure 5). Dothideomycetes and Microbotryomycetes dominate in sample A. Saccharomycetes and the sequences with no affiliation to any organisms in the public database (Unassigned) dominate in sample B. Dothideomycetes dominates in sample 1. Dothideomycetes and No-match dominate in sample 2. No-match dominates in sample 3 (Figure 5). NMDS ordination of the fungal community structure (abundance-based metrics) did show that the similarity of each sample is low, but there was some inclination between samples from the two sampling sites (Figure 6).

Open symbols represent samples from Kagoshima Bay and filled symbols represent samples from the Sao Paulo Ridge. The numbers beside the open symbols represent the sample depths from the seafloor (1: 0–5cmbsf, 2: 5–10cmbsf, 3: 10–15cmbsf). The alphabetical letters beside filled symbols represent A: sample collected from just below the whalebone, B: sample collected approximately 1 m away from the whalebone. Presence-absence based, 2D stress was 0.03.

## Discussion

### Fungal communities in marine sediments associated with whale-fall

A total of 107 OTUs were detected in this study. This is similar to the number of OTUs detected in deep-sea samples from the São Paulo Plateau, which also used the same study design (113 OTUs, 97% similarity: Nagano et al. 2017). However, this number is considerably less in comparison with the recently reported samples from several deep-sea environments analysed by use of the Illumina high-throughput sequencing system (four sediments, 420 OTUs, 97% similarity: Zhang et al. 2016; 9 sediments, 1742 OTUs, 98.5% similarity: Barone et al. 2018; 7 sediments, 723 OTUs, 97% similarity: Xu et al. 2018; 42 sediments, 890 OTUs, 97% similarity:, 2019; 29 sediments, 4421 OTUs, 97% similarity: Vargas-Gastelum et al. 2019). It is difficult to compare the fungal diversities analysed by different study designs (especially, sampling size and data processing methods). Also, the detection rate using different sequencing platforms should be clarified.

The most OTU-rich assignable phylum was Ascomycota (46%), followed by Basidiomycota (6%), which was consistent with most of the previous studies on fungal diversity in deep-sea sediments (Nagano and Nagahama 2012; Xu et al. 2014, 2018, 2019; Nagano et al. 2017; Vargas-Gastelum et al. 2019). Dothideomycetes (31%) was the most dominant OTUs in whale-fall sediments. Dothideomycetes are one of the common fungal classes to be reported from deep-sea environments (Nagahama and Nagano 2012). However, Eurotiomycetes and Sordariomycetes are generally the most dominant fungal classes in deep-sea sediment samples, previously reported by NGS (Zhang et al. 2016; Xu et al. 2018, 2019). Furthermore, the abundance of Dothideomycetes was quite low in the deep-sea sediment samples, which were taken from the Sao Paulo Plateau around the same time and analysed by the same methods (Nagano et al. 2017). In this study, domination by Dothideomycetes was shown in all the five samples examined. Higher domination was significantly present in samples taken closer to the whalebone. This suggests that domination by Dothideomycetes could be a characteristic feature of the whale-fall fungal composition. However, a larger number of samples with more suitable controls are needed to be investigated to claim this conclusion.

#### High detection rate of unidentified and unassigned fungal OTUs

The high detection of the unidentified fungi (21%) and unassigned (No-match)(16%) OTUs was revealed in the whale-fall environments in this study. It is known that a majority of deep-sea inhabiting fungal taxa still remain undescribed (Nagano et al. 2017) and deep-sea sediments can harbour a high number of novel fungal taxa (Barone et al. 2018). However, the detection rate of unassigned OTUs from deep-sea sediments remains much lower than 16%, for example, 2.4% in Zhang et al. (2016), 7.1% in Nagano et al. (2017), 0.03% in Xu et al. (2018), except Xu et al. (2019), which reported a relatively high detection rate (19.98%) of unassigned OTUs from the deep-sea hadal sediments of the Yap Trench. Also, it should be noted that there have been some studies reporting a high detection rate of unidentified fungi in shallow marine habitats (Jeffries et al. 2016; Picard 2017), but similarly this is not always the case. Therefore, there may be a hotspot for highly novel fungi (or other organisms) in marine environments including deep-sea, although it is not yet clear about the relationship between the high detection rate of unassigned OTUs and environmental factors. Furthermore, whale-fall chemosynthetic environments are certainly a unique organic- and sulphide-rich environment at the seafloor and are known to create remarkable habitats, as well as places of evolutionary novelty and biodiversity (Sumida et al. 2016). The same may apply for fungi as well.

#### High abundance of unidentified fungi

Another interesting feature of fungal communities in whale-fall environments was the high abundance of unidentified fungi. Within the top 10 detected most abundant OTUs, 4 OTUs were assigned to unidentified fungi and the second and the third most abundant OTUs were assigned to an unidentified fungus clone with very low coverage (Table 3). Due to the short length of sequence reads by Ion Torrent PGM and variability of ITS region, it was difficult to perform reliable phylogenic analysis for those unclassified sequences. However, some of these unidentified fungi are likely to be affiliated into early diverging fungi. For example, OTU2 showed 83% similarity (67% query coverage) with JX898611, which is suggested as a putative early-diverging fungal lineage in Zhang et al. (2014). OTU3 was assigned to uncultured basal lineage fungus clone (HM240101) by blastn search, but also showed 98% similarity (19% query coverage) with AB507855 and AB507858 that were reported as unknown sequences from methane cold-seep in Sagami Bay by Nagano et al. (2010). In the report, these sequences were grouped within the Kingdom Rhizaria, though with low support. Their phylogenetic position remained unclear, as there are no known organisms with similar sequences and these sequences could be early-diverging fungal lineages but also non-fungal or completely new lineages. OTU11 also showed 86% similarity (51% query coverage) with JX898611, which is suggested as putative early-diverging fungal lineage in Zhang et al. (2014). As these OTUs are abundant in the environment, they may play an important ecological role in whale-fall ecosystems, especially as OTU2 were detected in all the samples examined in this study. However, as OTU2 was detected at a distinctly higher rate in sample 3 (sample A: 27 sequences, sample B: 66, sample 1: 40, sample 2:322, sample 3:35599) and OTU3 was detected at a distinctly higher rate in sample B (sample A: 4, sample B: 22181, sample 1: 0, sample 2: 14, sample 3: 0), it may be interesting to investigate the correlation between their abundance and environmental features or the correlation between other organisms by co-analysis. It should be noted that unknown basal fungal lineages abundantly appeared from other deep-sea chemosynthetic environments, such as hydrocarbon seep sediments (Nagahama et al. 2011), hydrothermal ecosystems (Le Calvez et al. 2009) and anoxic sediment around a submarine caldera (Takishita et al. 2005). These unknown basal fungal lineages may have adapted and play a key role in those unique deep-sea chemosynthetic environments. Later, some of these unknown basal fungal lineages reported from deep-sea environments were affiliated within a novel fungal phylum, Cryptomycota (Jones et al. 2011) and NCLC (Novel-Chytrid-Like-Clade) groups (Richards et al. 2015). The presence of Cryptomycota and unknown basal fungal lineages, such as NCLC are more recognised and have been reported in recent years from many environments, including land, freshwater, sea ice, shallow to deep marine, with more extensive reporting from aquatic environments (both fresh and marine) (Jones et al. 2011; Lazarus and James 2015; Ishii et al. 2015; Richards et al. 2015; Comeau et al. 2016; Hassett and Gradinger 2016; Picard 2017; Rojas-Jimenez et al. 2017; Wang et al. 2018; Lepere et al. 2019). Picard (2017) reported that marine benthic sediments harboured high proportion of novel sequences, which were assigned to early-diverging fungal groups and could not be assigned beyond phylum with statistical support, suggesting they belong to unknown lineages.

Richards et al. (2015) suggested that some unknown basal fungal groups, such as NCLC1, have only been detected in marine environments, which encompasses a significant marine radiation of this group. It is difficult to determine if unidentified and unassigned ITS sequences from our study are also affiliated into these unknown basal fungal groups, such as Cryptomycota and NCLC. Although it has been suggested as more advantageous to target the ITS regions for PCR analysis in detecting fungal DNAs in deep-sea sediments (Nagano et al. 2010), targeting the more conserved 18S r RNA and 28S r RNA regions may be a more effective method for locating DNA libraries with many unknown sequences. Further investigation on whale-fall fungal communities by targeting the 18S r RNA and 28S rRNA will be needed to reveal the cryptic fungal communities detected in this study. Also, revealing the ITS sequences of those basal fungal groups and building up the public database will help the taxonomic annotation of unidentified fungi. Some of these works may be available to do by employing a walking PCR method on unknown ITS sequences and detecting known 18S rRNA and 28S rRNA sequences. Furthermore, as Hassett et al. (2020) reported that only 50% of marine fungal taxa have a nucleotide sequence and only ~12% are represented in NCBI’s RefSeq database, it is essential to expand the collection of reference sequence data for a better understanding of the ecology of marine fungi.

#### High abundance of DSF-group1

DSF-group1 was first described by Nagano et al. (2010) and it has been recognised as uncultured taxa related to *Metschnikowia/Candida*, frequently and widely detected from deep-sea sediments, e.g. hydrocarbon seeps in Sagami Bay (Nagano et al. 2010; Nagahama et al. 2011), Gulf of Mexico (Thaler et al. 2012; Vargas-Gastelum et al. 2019), the Izu-Ogasawara Trench (Nagano et al. 2010) and the Mariana Trench (Xu et al. 2014) of the Pacific Ocean and the Chinese Seas (Li et al. 2016). Generally, this group is found in oxygen-depleted deep-sea sediments and this group was detected also in the whale-fall sediments examined in this study (OTU4, OTU34, OTU84). OTU4 was detected as the 4^th^ abundant OTU and was detected in all the five samples (sample A: 441 sequences, sample B: 17467, sample 1: 330, sample 2:220, sample 3:2613). Our results provide further evidence to support that DSF-Group1 is commonly present in deep-sea environments and more abundantly present in oxygen-depleted deep-sea environments. Although this group has been reported often from deep-sea sediment samples, there is no successful culture strain yet. It would be interesting to know their physiological features.

### Fungal community difference between each sample

#### Highest fungal diversity in the sample 2 (sediment 5–10 cm below the whale bone)

The highest fungal diversity was detected in sediment 5–10 cm below the whalebone rather than the surface sediment. This may be related to the chemical environment of the whale-fall sediments. It has been reported that the total organic carbon (TOC) was elevated above background levels from the sediment surface to 11 cm in depth with a peak of 6 cm (Treude et al. 2009). The strong correlation between the TOC and the richness of fungal diversity has been reported previously (Orsi et al. 2013; Tedersoo et al. 2014) and was consistent with the results from this study.

#### Unidentified and unassigned fungi are more abundant in sediments located away from the whale bone

The OTUs composition of fungal communities at the class level throughout all the five samples showed a similar pattern (Figure 3). This result was very interesting, as the samples were collected in completely different settings, geologically (Brazil, Japan) and physiologically, e.g., depths (4204 m, 101 m). It is suggested that fungal communities are more sensitive to the chemical and biological environments created by whale-falls than by geological or depth settings. There have been some studies reporting the absence of significant correlation between fungal composition and depth (Nagano et al. 2017; Vargas-Gastelum et al. 2019), and our results were consistent with their analysis.

The most interesting result from this study was that the abundance of each fungal class showed a significant difference between each sample, but showed some interesting inclination (Figure 5), that is a higher abundance of unknown fungal communities (unidentified fungi and unassigned fungi), which are likely related to basal fungi, and were more abundant in sediments deeper from the surface, as well as in sediment farther from the whalebone. In contrast, Ascomycetous fungi and Basidiomycetous yeasts are dominated in the sediments just below the whalebone. This may be explained by Ascomycetous fungi, such as Dothidiomycetes and Basidiomycetous yeasts, such as Mycrobotryomycetes being saprophytic fungi and degrading the remaining whale carcases, which are more abundant in the sediments in contact with whalebone. Although the reason for this inclination cannot be determined from our experiments, it is a very interesting phenomenon and further investigation will be needed to elucidate the relationship between cryptic basal fungal communities and whale-fall chemosynthetic environments and their ecological role.

## Conclusions

This study suggests that unknown fungal communities exist abundantly in unique whale-fall chemosynthetic ecosystems from two investigated sites, one naturally occurring at 4200 m water depth in South Atlantic Ocean, and one artificially immersed at 100 m water depth in Kagoshima Bay, Japan. Interestingly, unidentified and unassigned fungi, which are implicated to be early diverging fungi, were more abundant in the sediments not directly in contact with whalebone. As a short ITS region, which was used in this study, it is not sufficient to perform phylogenetic analysis. Further study targeting a longer and more conserved region, such as 18S rRNA and 28S rRNA, will be needed to reveal unknown fungal community in whale-fall ecosystems. Although our knowledge on fungal diversity in deep-sea environments has significantly increased in the last decade, it is still fragmentary and limited within the vast expanse of the deep sea. The true diversity and ecological role of deep-sea fungi remains unclear, especially for those unknown basal fungal groups frequently detected from these environments. Thus, it is important to understand the ecological and physiological significance of these fungi, especially those that are possibly endemic in deep-sea environments. Efforts to isolate culture strains of these unknown basal fungal groups should be made as well. This will help provide key insights into the phylogenetic histories of fungi and their mechanisms of adaptation to extreme environments, and should provide a better understanding of unique deep-sea ecosystems.
